# Oral HPV Dynamics in MSM Living with HIV in the Nine-Valent HPV Vaccination Era

**DOI:** 10.3390/vaccines14070589

**Published:** 2026-07-01

**Authors:** Verdiana Zulian, Martina De Sanctis, Silvia Pauciullo, Roberta Sciamanna, Paola Del Porto, Anna Rosa Garbuglia

**Affiliations:** 1 Laboratory of Virology, National Institute for Infectious Diseases “Lazzaro Spallanzani” (IRCCS), 00149 Rome, Italy; martina.desanctis@inmi.it (M.D.S.); silvia.pauciullo@inmi.it (S.P.); roberta.sciamanna@inmi.it (R.S.); annarosa.garbuglia@inmi.it (A.R.G.); 2 Department of Biology and Biotechnology “Charles Darwin”, Sapienza University of Rome, 00100 Rome, Italy; paola.delporto@uniroma1.it

**Keywords:** human papillomavirus, vaccine, nine-valent, MSM, PLWH, viral persistence, HPV16

## Abstract

**Background/Objectives**: Oral human papillomavirus (HPV) infection is emerging as a key driver of HPV-associated oropharyngeal cancer, especially in high-risk groups such as men who have sex with men (MSM) living with HIV (PLWH). However, evidence on oral HPV persistence and the impact of nine-valent HPV vaccination in adults remains limited. We conducted a prospective longitudinal study including 76 MSM PLWH, of whom 64 were nine-valent HPV-vaccinated and 12 unvaccinated. **Methods**: Oral rinse samples were collected at baseline (T0) and after 6 months (T6). HPV DNA detection and genotyping were performed using the Allplex™ HPV28 assay. Oral HPV dynamics (persistence, clearance, and incidence) were assessed for high-risk (HR) HPV, low-risk (LR) HPV, and vaccine-type HPV genotypes. **Results**: Baseline oral HPV prevalence was high (59.2%), with HR HPV detected in 43.4% of participants. HPV16 was the most frequent genotype at both T0 and T6. Among participants HPV-positive at baseline, persistence of HPV DNA was high and similar regardless of vaccination status (77.8%). However, incident vaccine-type oral HPV infection was significantly lower among vaccinated individuals than unvaccinated participants (6.3% vs. 33.3%; OR 0.13, 95% CI: 0.03–0.71; *p* = 0.0441). Finally, reporting ≥10 sexual partners in the previous year was significantly associated with baseline oral HPV positivity (*p* = 0.0298). **Conclusions**: In MSM PLWH, oral HPV infection is highly prevalent and persistent, underscoring that it may represent a reservoir for HPV-related oropharyngeal disease. In our small observational cohort, nine-valent HPV vaccination was associated with lower incident detection of vaccine-type oral HPV, supporting targeted vaccination and oral HPV surveillance in high-risk adult populations, while highlighting the need for larger longitudinal studies to confirm these findings and better define the magnitude and durability of vaccine-associated protection at the oral site.

## 1. Introduction

Human papillomavirus (HPV) is increasingly recognized as a major etiological factor in oropharyngeal cancer (OPC). Globally, approximately 31% of OPC cases are attributable to HPV infection [1]. However, this proportion appears to be rising in several countries, particularly in the United States, where HPV may account for up to 90% of OPC cases [2,3]. In some settings, the burden of HPV-positive OPC now exceeds that historically associated with tobacco and alcohol exposure, which were considered the main risk factors for this malignancy until the 1990s [4]. Notably, in the United States, the annual number of HPV-associated OPC cases in men surpassed that of cervical cancer cases in women in 2016 [5,6,7].

OPC incidence is substantially higher in men than in women, with a male-to-female rate ratio of approximately 4.8 [8,9,10]. Among HPV-associated OPCs, HPV16 is by far the predominant genotype, whereas other high-risk types, including HPV18, HPV35, and HPV45, are detected less frequently [11]. HIV-related immunosuppression may further increase susceptibility to oral HPV infection by impairing viral clearance [12]. Consistent with this, a systematic review estimated that the prevalence of oral high-risk HPV among HIV-positive men who have sex with men (MSM) is approximately fourfold higher than that observed in the general population and about twofold higher than that reported in MSM without HIV infection [13,14]. In addition, people living with HIV (PLWH) have been shown to carry a higher prevalence of HPV infection at baseline and to exhibit a longer time to viral clearance than HIV-negative individuals [15].

The mechanisms underlying HPV-driven OPC remain incompletely understood. In contrast to anogenital HPV-related malignancies, no well-defined precursor lesion has yet been identified for OPC. Nevertheless, viral persistence is considered a key event in malignant transformation. In a recent observational study of men, only 24.3% of oral HPV infections persisted over time, and older age was the only factor associated with persistent infection [16]. However, data on persistence in vulnerable populations, including vaccinated MSM PLWH, are still lacking.

Prophylactic HPV vaccination has proven effective in reducing the burden of HPV-related disease, particularly at the cervical and anal sites. The currently available nine-valent vaccine (Gardasil 9) targets seven high-risk (HR) HPV types (HPV16, 18, 31, 33, 45, 52, and 58) and two low-risk (LR) types (HPV6 and 11), thereby covering the genotypes responsible for the large majority of HPV-related cancers [17,18,19], including those implicated in the majority of HPV-positive OPCs [20,21]. Notably, emerging evidence further suggests that HPV vaccination is associated with reduced odds of developing OPC [22]. However, evidence on the effect of HPV vaccination in preventing OPC remains limited.

Vaccine-induced antibody responses are generally stronger and more durable when immunization is administered at younger ages, before sexual debut [23,24]. PLWH, in whom multiple HPV infections are more common and viral persistence is more likely, may mount a weaker response to vaccination [25].

In Italy, the National Immunization Plan 2023–2025 prioritizes the prevention of HPV-related cancers through an equitable and standardized vaccination strategy across regions [26]. The HPV vaccine is offered free of charge to girls and boys at 11 years of age, with a 2-dose schedule for individuals initiating vaccination before 15 years of age and a 3-dose schedule for those starting at 15 years of age or older. Catch-up vaccination is also offered for women up to at least 26 years of age and for men up to at least 18 years of age. In addition, expanded vaccination access is recommended for several high-risk groups, including individuals treated for high-grade cervical lesions (CIN2+), MSM, and PLWH. Despite these recommendations, evidence on the effect of HPV vaccination on oral HPV persistence, particularly among MSM PLWH, remains scarce.

In this study, we assessed longitudinal oral HPV dynamics in nine-valent HPV-vaccinated and unvaccinated MSM PLWH over a 6-month follow-up period.

## 2. Materials and Methods

### 2.1. Study Population

Participants attending the Dermatological Unit of the National Institute for Infectious Diseases (INMI) “Lazzaro Spallanzani”-IRCCS in Rome, Italy, were invited to participate in this study. The study was approved by the local Ethics Committee (ethical approval number 75/2024), and all participants provided written informed consent.

Eligibility criteria were as follows: (i) HIV infection; (ii) age ≥18 years; (iii) receipt of antiretroviral therapy (ART) for at least 12 months; and (iv) no history of mucosal head and neck cancer or clinically evident oral lesions within the previous 6 months.

At enrollment, participants completed a standardized questionnaire collecting information on lifestyle factors (tobacco use, alcohol consumption, and recreational drug use), HPV vaccination history (previous vaccination, age at vaccination, vaccine formulation, and number of doses), self-reported sexual orientation, and sexual behavior (number of sexual partners in the previous year and oral sex practices). HPV vaccination history was primarily determined from participant self-report through the standardized questionnaire; when questionnaire information was incomplete, unavailable, or uncertain, vaccination data were obtained from the treating physician and/or available clinical documentation.

Participants were classified as HPV-vaccinated if they had completed the full three-dose schedule of the nine-valent HPV vaccine at least two years before baseline oral sampling.

A medical history and physical examination were performed at baseline (T0) and 6-month follow-up (T6). Immuno-virological parameters, including CD4+ T-cell count and HIV RNA viral load, were retrieved from the institutional virology laboratory records.

### 2.2. HPV Infection Definition

Oral HPV prevalence and infection dynamics were assessed at T0 and at T6, both at the participant level and according to HPV genotype (i.e., genotype-specific level) [16].

At the participant level, HPV positivity (any HPV) was defined as the detection of at least one HPV genotype included in the Seegene Allplex™ HPV28 assay (Seegene, Seoul, Republic of Korea) in the oral sample at a given visit. High-risk HPV (HR-HPV) positivity was defined as the detection of at least one genotype classified as high-risk in this study (HPV16, HPV18, HPV31, HPV33, HPV35, HPV39, HPV45, HPV51, HPV52, HPV56, HPV58, HPV59, HPV66, HPV68, HPV73, and HPV82). Low-risk HPV (LR-HPV) positivity was defined as the detection of at least one genotype classified as low-risk (HPV6, HPV11, HPV26, HPV40, HPV42, HPV43, HPV44, HPV53, HPV54, HPV61, HPV69, HPV70, and HPV74). Vaccine-type HPV positivity was defined as the detection of at least one genotype included in the nine-valent vaccine (HPV6, HPV11, HPV16, HPV18, HPV31, HPV33, HPV45, HPV52, and HPV58).

HPV infection dynamics between T0 and T6 were assessed at the participant level and, where applicable, at the genotype-specific level.

At the participant level, for each HPV definition (any HPV, HR-HPV, LR-HPV, and vaccine-type HPV), outcomes were classified as persistence, clearance, incidence, and remaining negative. For HR-HPV, LR-HPV, and vaccine-type HPV, participant-level persistence was defined as category-level persistence, as positivity at both T0 and T6 required the presence of at least one genotype belonging to the same category, not necessarily the same genotype across visits. Therefore, category-level persistence does not necessarily indicate biological persistence of an identical genotype.

At the genotype-specific level, genotype-specific persistence was defined as the detection of the same HPV genotype at both T0 and T6. Genotype-specific clearance was defined as positivity for a given genotype at T0 followed by negativity for that genotype at T6, and genotype-specific incidence as negativity at T0 followed by positivity at T6.

Participants were also stratified according to HPV vaccination status (vaccinated vs. unvaccinated) and age group (≥45 years vs. <45 years). Finally, infections were classified as single or multiple based on the number of HPV genotypes detected at each visit, with multiple infection defined as detection of more than one genotype in the same sample.

### 2.3. Biospecimens, DNA Extraction, and HPV DNA Detection

Oral rinse specimens were collected at T0 and T6. Briefly, participants performed a 10 s oral rinse followed by at least a 10 s gargle using 15–20 mL of mineral water and then expectorated into a sterile container. Specimens were stored at 2–8 °C and delivered on the same day at the Virology Laboratory of INMI “Lazzaro Spallanzani”-IRCCS.

Samples were concentrated by centrifugation (3000× *g* for 10 min) to obtain a pellet, which was resuspended in 2 mL phosphate-buffered saline (PBS). The suspension was divided into two aliquots: one aliquot (1 mL) was used for nucleic acid extraction, and the second aliquot was stored at −80 °C.

Nucleic acids were extracted using an automated platform (Seegene STARlet; Seoul, Republic of Korea). HPV DNA detection and genotyping were performed using the Allplex™ HPV28 assay. The assay detects 28 HPV genotypes, including 19 high-risk types (HPV16, 18, 26, 31, 33, 35, 39, 45, 51, 52, 53, 56, 58, 59, 66, 68, 69, 73, and 82) and 9 low-risk types (HPV6, 11, 40, 42, 43, 44, 54, 61, and 70). The method is based on tagging oligonucleotide cleavage and extension technology combined with multiple detection temperature (MuDT™) technology, enabling simultaneous genotype identification and generation of genotype-specific cycle threshold (Ct) values. Samples with genotype-specific Ct values ≤43 were considered HPV-positive.

For viral load proxy analyses, genotype-specific Ct values were normalized using the assay internal control, beta-globin housekeeping gene, to derive ΔCt values (Ct_HPV–Ct_internal control) [27]. For genotype-persistent detections, changes over time were assessed using ΔΔCt, calculated as ΔCt(T6)−ΔCt(T0); positive ΔΔCt values were interpreted as a decrease in viral load, whereas negative values were interpreted as an increase.

### 2.4. Statistical Analysis

Continuous variables are reported as median (interquartile range, IQR) or range, and categorical variables as frequencies and percentages (*n*, %). Univariable analyses were performed to assess associations between clinical and behavioral variables and HPV-related outcomes. Associations between categorical variables were analyzed using Fisher’s exact test, and comparisons of continuous variables between groups were performed using the Mann–Whitney U test. Correlations between continuous variables were assessed using Spearman’s rank correlation coefficient. Effect estimates are reported as odds ratios (ORs) with 95% confidence intervals (95% CIs); relative risks (RRs) were calculated for selected comparisons to aid interpretability. All tests were two-sided, and *p* < 0.05 was considered statistically significant. Statistical analyses were performed using GraphPad Prism version 10 (GraphPad Software, San Diego, CA, USA).

Given the limited sample size, particularly the small number of unvaccinated participants, the small denominators in several subgroup analyses, and missing data for some self-reported behavioral variables, the statistical power to detect differences was limited. Therefore, the results should be interpreted with caution. In addition, no multivariable regression analyses were performed because of the limited sample size and low number of outcome events; thus, potential confounding by age, sexual behavior, baseline HPV status, and other behavioral variables could not be fully accounted for.

## 3. Results

### 3.1. Characteristics and Description of the Study Population

A total of 76 MSM PLWH were included in the study, comprising HPV-vaccinated (*n* = 64) and HPV-unvaccinated participants (*n* = 12). The median age at baseline was 51 years (range 30–70), with 68.4% (*n* = 52) aged ≥45 years. Most participants were Italian (89.5%), whereas 10.5% had other nationalities; the proportion of participants with other nationalities was numerically higher among unvaccinated than vaccinated individuals (25.0% vs. 7.8%) (Table 1). Among vaccinated, 32.8% (*n* = 21) aged <45 years. The median age at vaccine initiation was 47 years (range 26–68). All vaccinated participants had completed the full three-dose schedule of the nine-valent HPV vaccine (Gardasil 9) at least 2 years before sample collection. All participants were sexually active at the time of sampling.

At T0, 59.2% of participants were oral HPV-positive. HPV positivity was higher in the unvaccinated group (75.0%) than in the vaccinated group (56.3%). Multiple HPV infections were detected in 25.0% of the cohort (23.4% in vaccinated vs. 33.3% in unvaccinated groups). Overall, 25.0% were positive for at least one nine-valent vaccine-type genotype and 43.4% for at least one high-risk genotype, with no statistically significant differences between vaccinated and unvaccinated participants (Table 1). However, these baseline comparisons, particularly the numerically higher oral HPV positivity in unvaccinated participants, should be interpreted cautiously because of the very small size of the unvaccinated group. Therefore, the lack of statistical significance should not be interpreted as evidence of comparability between groups.

Regarding HIV-related parameters, the duration of ART exposure ranged from 7 to 25 years, and HIV RNA was undetectable in 64.5% of participants. Detectable HIV RNA was observed in 34.2% with <30 copies/mL and in 1.3% with >30 copies/mL, with a similar distribution across vaccination groups (Table 1). The overall median CD4+ T-cell count was 710.0 cells/µL (IQR: 504.0–895.0). Most participants had CD4+ ≥500 cells/µL (77.7%, *n* = 59), while 19.7% (*n* = 15) had 201–499 cells/µL and 2.6% (*n* = 2) had ≤200 cells/µL. Median CD4+ T-cell counts were similar in HPV-vaccinated and HPV-unvaccinated participants (713.0 cells/µL [IQR 515.0–889.5] vs. 672.0 cells/µL [IQR 288.5–884.0], respectively). Most vaccinated participants had CD4+ ≥500 cells/µL (79.6%, *n* = 51), while 18.8% (*n* = 12) had 201–499 cells/µL and 1.6% (*n* = 1) had ≤200 cells/µL. In the unvaccinated group, 66.7% (*n* = 8) had CD4+ ≥500 cells/µL, 25.0% (*n* = 3) had 201–499 cells/µL, and 8.3% (*n* = 1) had ≤200 cells/µL; the distribution of CD4+ categories did not differ significantly by vaccination status (*p* = 0.5331) (Table 1).

Univariable analyses were performed to identify factors associated with baseline oral HPV positivity (Table 2). Baseline oral HPV positivity was not significantly associated with age category, CD4+ T-cell count, HIV RNA detectability, or HPV vaccination status.

Sexual behavior emerged as the main determinant of baseline oral HPV detection: reporting ≥10 sexual partners in the previous year was associated with a 55% higher probability of oral HPV positivity (RR 1.55, 95% CI: 1.09–2.30), and higher odds (OR 4.09, 95% CI: 1.33–11.85; *p* = 0.0298) compared with reporting fewer partners. No statistically significant associations were observed for the other behavioral variables evaluated (Table 2).

### 3.2. Prevalence of Oral HPV Genotypes at Baseline and Follow-Up

#### 3.2.1. Prevalence of Oral HPV Genotypes in Overall Population

At baseline, HPV16 was the most prevalent HR-HPV genotype in the overall population (10.5%), followed by HPV73 (7.9%), HPV56 and HPV66 (6.6% each), and HPV39 (5.3%). Lower frequencies were observed for HPV33, HPV35, HPV58, and HPV82 (3.9% each), whereas HPV18, HPV51, HPV52, and HPV59 were detected less frequently (≤2.6%) (Table S1). At T6, HR-HPV genotypes continued to predominate in the overall population (Table S2). HPV16 was again the most frequently detected genotype (11.8%), followed by HPV66 (7.9%) and HPV33, HPV39, and HPV58 (6.6% each). HPV18, HPV35, HPV51, HPV59, HPV68, HPV73, and HPV82 were detected at lower frequencies (≤3.9%), whereas HPV31, HPV45, HPV52, and HPV56 were not observed at follow-up.

#### 3.2.2. Prevalence of Oral HPV Genotypes by Age

Genotype distribution differed by age group and sampling time. In participants younger than 45 years, HPV61 (12.5%) was the leading genotype at baseline, while HPV16 (12.5%) became the most prevalent genotype at T6, with a broader distribution of additional HR- and LR types detected at lower frequencies. Among participants aged 45 years or older, HPV16 remained one of the predominant genotypes at both visits (11.5% each), accompanied by HPV53 at baseline (11.5%) and HPV44 at T6 (11.5%); HPV61, HPV66, HPV73, HPV33, and HPV53 were also frequently detected in this age group.

#### 3.2.3. Prevalence of Oral HPV Genotypes by Vaccination Status

When stratified by vaccination status, HPV16 remained the most frequent HR-HPV genotype among vaccinated participants (10.9%), followed by HPV39, HPV56, HPV66, and HPV73 (6.3% each) at baseline (Table S1). Among vaccine-targeted genotypes, HPV33 and HPV58 were detected in 4.7% and 3.1% of vaccinated individuals, respectively, whereas HPV18, HPV31, and HPV45 were not observed. In the unvaccinated group, HR-HPV genotypes showed a more restricted distribution, with HPV35 and HPV73 being the most frequent types (16.7% each), followed by HPV16, HPV18, HPV56, HPV58, HPV59, and HPV66 (8.3% each). HPV33, HPV39, HPV52, and HPV82 were not detected among unvaccinated participants. Regarding LR-HPV genotypes, HPV61 (10.5%) and HPV53 (9.2%) were the most frequently detected types overall. Among vaccinated participants, HPV53 (10.9%) and HPV61 (7.8%) predominated, whereas HPV61 was the most common LR-HPV genotype in the unvaccinated group (25.0%), followed by HPV44 (8.3%). Other LR-HPV genotypes were detected sporadically or were absent across groups (Table S1).

At T6 (Table S2), HPV16 remained the leading HR-HPV genotype among vaccinated individuals (12.5%). Other frequently detected HR-HPV genotypes in this group included HPV39 and HPV66 (7.8% each), HPV33 and HPV58 (6.3% each), and HPV82 (4.7%). In the unvaccinated group, HR-HPV detection was limited to fewer genotypes, with HPV35 and HPV73 being the most frequent types (16.7% each), followed by HPV16, HPV18, HPV58, HPV59, and HPV66 (8.3% each).

LR-HPV genotypes were less frequently detected at follow-up. Overall, HPV44 (7.9%), HPV61 (6.6%), and HPV53 (5.3%) were the most common LR types. Among vaccinated participants, HPV44 and HPV53 were detected most frequently (6.3% each), followed by HPV11, HPV43, and HPV61 (4.7% each). In unvaccinated participants, LR-HPV detection was limited to HPV6, HPV44, and HPV61, each observed in 16.7% of individuals.

#### 3.2.4. Oral HPV Genotype-Specific Persistence and Incidence

Overall, 19 HPV genotypes (HPV6, HPV11, HPV16, HPV18, HPV33, HPV35, HPV39, HPV43, HPV44, HPV51, HPV53, HPV58, HPV59, HPV61, HPV66, HPV69, HPV70, HPV73, and HPV82) showed genotype-specific persistence between T0 and T6 in the study cohort (Figure S1). Among persistent genotype detections, HPV16 was the most frequently observed genotype (18.2%), followed by HPV61 (9.1%). Persistence was also observed for HPV33, HPV39, HPV53, HPV58, and HPV73 (6.8% each).

When stratified by HPV vaccination status, persistent genotype detections were observed in both vaccinated and unvaccinated participants. Among vaccinated participants HPV16 was the most frequently persistent genotype (29.2%), followed by HPV33, HPV39, and HPV53 (12.5% each). In contrast, persistent infections among unvaccinated participants involved a more restricted set of genotypes, with HPV35, HPV61, and HPV73 being the most frequently observed persistent types (28.6% each).

**Figure 1:**
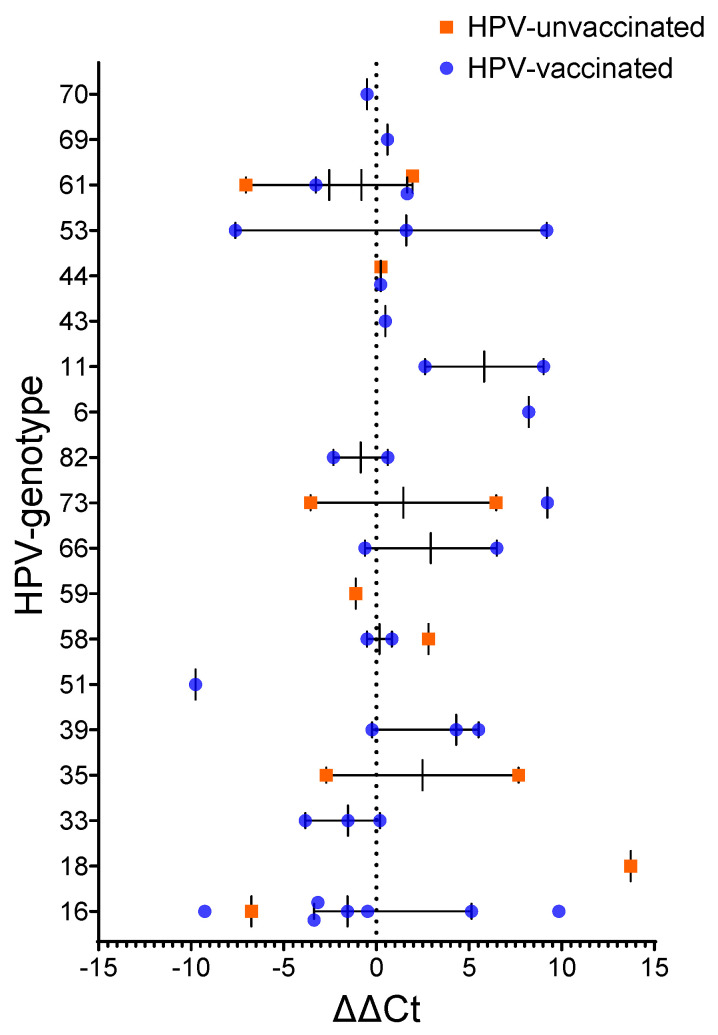
Comparison of ΔΔCt values for persistent oral HPV genotypes between vaccinated and unvaccinated individuals. ΔΔCt values (T6–T0) for persistent oral HPV genotypes, stratified by vaccination status. Positive ΔΔCt values indicate a decrease in viral load, while negative values indicate an increase. Points represent individual genotype-specific infections; horizontal lines indicate median and interquartile range (IQR). The number of observations for each persistent genotype was as follows: HPV16 (*n* = 8), HPV18 (*n* = 1), HPV33 (*n* = 3), HPV35 (*n* = 2), HPV39 (*n* = 3), HPV6 (*n* = 1), HPV11 (*n* = 2), HPV43 (*n* = 1), HPV44 (*n* = 2), HPV51 (*n* = 1), HPV53 (*n* = 3), HPV58 (*n* = 3), HPV59 (*n* = 1), HPV61 (*n* = 4), HPV66 (*n* = 2), HPV69 (*n* = 1), HPV70 (*n* = 1), HPV73 (*n* = 3), and HPV82 (*n* = 2).

ΔΔCt values were calculated for the 19 genotype-persistent HPV detections and demonstrated substantial inter-individual variability, with an overall median ΔΔCt value of 0.225 (IQR: −2.515 to 3.558). Median ΔΔCt values were comparable between vaccinated and unvaccinated participants (0.21 vs. 0.25, respectively), with no statistically significant difference between groups (*p* = 0.8810) (Figure 1).

When genotype-specific ΔΔCt values were considered, distinct patterns emerged according to vaccination status (Figure 1). Among vaccinated participants, HPV16 (median −2.36), HPV33 (median −1.54), and HPV82 (median −0.85) showed median ΔΔCt values below zero, suggesting stable or increasing viral loads between T0 and T6. Conversely, HPV11 (median 5.82), HPV39 (median 4.31), HPV53 (median 1.61), and HPV66 (median 2.94) showed positive median ΔΔCt values, consistent with a reduction in viral load over time. Among unvaccinated participants, HPV61 was associated with negative ΔΔCt values (median −2.55), whereas HPV73 showed positive ΔΔCt values (median 1.46).

Finally, among genotypes detected at baseline, ΔCt values at T0 were slightly higher in infections that subsequently cleared than in those that persisted (median 10.40 vs. 9.29), although this difference was not statistically significant (*p* = 0.167) (Figure S2).

#### 3.2.5. Participant-Level Oral HPV Persistence and Incidence

Considering that HPV infection dynamics between T0 and T6 were assessed at the participant level, persistence of any HPV infection (Table 3) was comparable between vaccinated and unvaccinated participants (77.8% in both groups).

**Figure 2:**
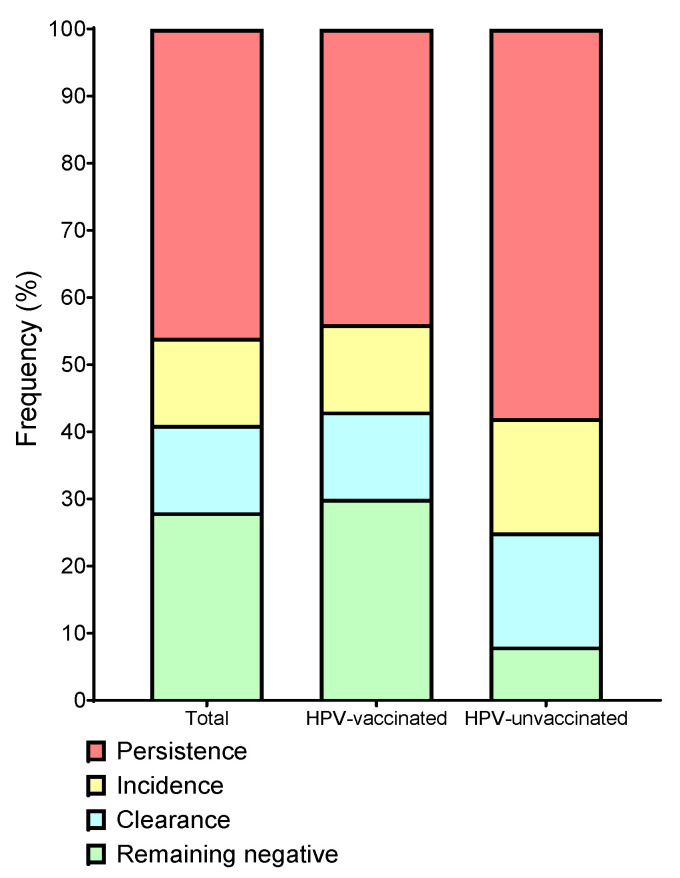
Participant-level distribution of any oral HPV infection outcomes by vaccination status. Bars show the percentage of participants in each group (total cohort, HPV-vaccinated, HPV-unvaccinated) classified as having HPV persistence (red), incidence (yellow), clearance (blue), or remaining negative (green) between baseline (T0) and 6-month follow-up (T6). Percentages are calculated using the total number of participants in each group as the denominator.

By contrast, participants who were HPV-negative at T0, incident oral HPV infection at T6 was observed in both vaccinated and unvaccinated individuals, with a lower incidence in the vaccinated group (28.6%) compared with unvaccinated participants (66.7%), although this difference did not reach statistical significance (OR 0.20, 95% CI: 0.02–2.53; *p* = 0.2369). Finally, most baseline-negative participants in the vaccinated group remained HPV-negative at both visits (71.4%) (Table 3). Participant-level outcome distributions are shown in Figure 2.

With respect to HR-HPV genotypes, persistence at participant level remained high in both groups and was slightly higher among unvaccinated participants (69.2% and 85.7%, respectively; *p* = 0.6418) (Table 4). In participants who were HR-HPV-negative at T0, incident HR-HPV infection at T6 was detected in both study groups, occurring in 18.4% of vaccinated participants and 40.0% of unvaccinated participants, with no statistically significant difference between groups (OR 0.34, 95% CI: 0.06–2.25; *p* = 0.2773). Most baseline HR-HPV–negative participants remained HR-HPV-negative at both visits, particularly in the vaccinated group (81.6% vs. 60.0%) (Table 4).

Regarding LR-HPV genotypes, both clearance and persistence at T6 were comparable between vaccinated and unvaccinated participants who were LR-HPV-positive at T0. Clearance occurred in 42.1% of vaccinated individuals and 33.3% of unvaccinated individuals (OR 1.46, 95% CI: 0.15–23.43; *p* > 0.9999), while persistence was observed in 57.9% and 66.7% of participants, respectively (Table 4). Similarly, among participants who were LR-HPV-negative at baseline, LR-HPV incidence at T6 did not differ between groups, occurring in 13.3% of vaccinated and 11.1% of unvaccinated individuals (OR 1.23, 95% CI: 0.14–15.73; *p* > 0.9999), and the majority remained LR-HPV-negative at both visits in both groups (86.7% vs. 88.9%) (Table 4).

Analyses were therefore restricted to HPV genotypes targeted by the nine-valent vaccine (HPV6, HPV11, HPV16, HPV18, HPV31, HPV33, HPV45, HPV52, and HPV58).

Among participants who were positive for at least one vaccine-type HPV genotype at T0, clearance at T6 was infrequently observed and occurred only among vaccinated participants (12.5%), whereas no clearance was observed among unvaccinated participants; persistence remained high in both groups (87.5% in vaccinated vs. 100% in unvaccinated participants) (Table 5).

**Figure 3:**
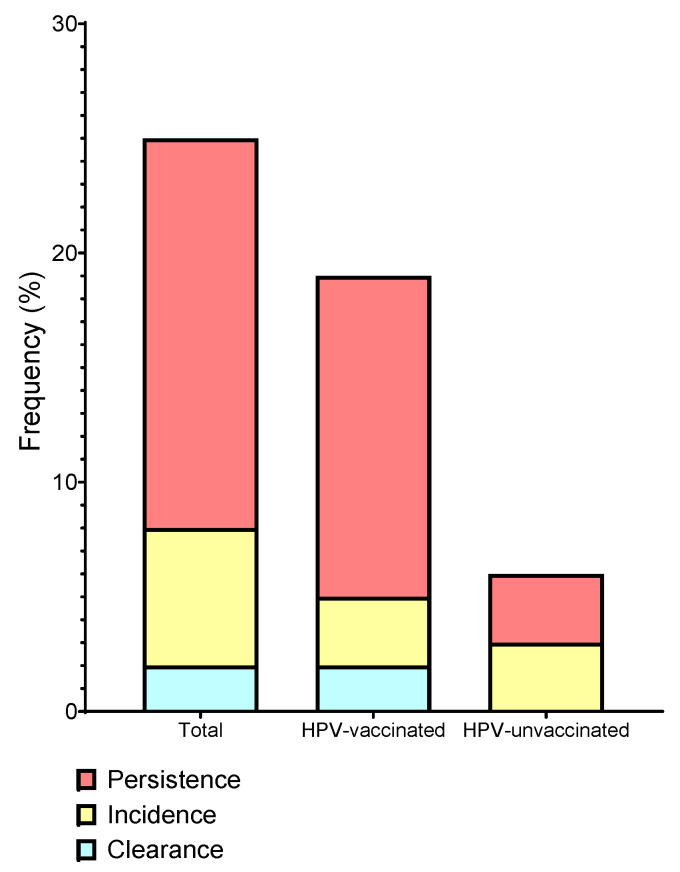
Participant-level distribution of vaccine-type HPV infection outcomes in oral samples by vaccination status. Bars show the percentage of participants in each group (total cohort, vaccinated, unvaccinated) who showed vaccine-type HPV persistence (red), incidence (yellow), or clearance (blue) between baseline (T0) and at 6-month follow-up (T6). Percentages are calculated using the total number of participants in each group as the denominator.

In contrast, among participants who were vaccine type HPV-negative at baseline, incident vaccine type HPV infection at follow up was significantly less frequent in vaccinated individuals compared with unvaccinated participants (6.3% vs. 33.3%; OR 0.13, 95% CI: 0.03–0.71; *p* = 0.0441). Consistently, a higher proportion of vaccinated participants remained negative for vaccine type HPV at both visits compared with unvaccinated participants (93.7% vs. 66.7%) (Table 5). Participant level outcome distributions are shown in Figure 3. However, this comparison was based on a small number of incident events and a very limited number of baseline-negative unvaccinated participants and should therefore be interpreted with caution.

### 3.3. Longitudinal Oral HPV Infection Dynamics by Age

In age-stratified analyses, participant-level infection dynamics were evaluated using the same definitions described above; therefore, persistence for any-HPV, HR-HPV, LR-HPV, and vaccine-type HPV refers to category-level persistence.

#### 3.3.1. Dynamics of Any Oral HPV Infection by Age

In participants who were HPV-positive at T0, persistence at participant level remained high in both groups (78.1% in participants aged ≥45 years vs. 76.9% in those aged <45 years) (Table S3).

Conversely, when considering participants who were HPV-negative at T0, incident oral HPV infection at T6 was observed in both age groups. Incidence was higher in participants aged ≥45 years compared with those younger than 45 years (40.0% vs. 18.2%), although this difference was not statistically significant (OR 3.00, 95% CI: 0.56–16.19; *p* = 0.2617). Consistently, most baseline-negative individuals remained HPV-negative at both visits, with a higher proportion observed in participants aged <45 years than in those aged ≥45 years (81.8% vs. 60.0%) (Table S3).

#### 3.3.2. Dynamics of High-Risk and Low-Risk Oral HPV Infection by Age

When considering HR-HPV genotypes, longitudinal patterns between T0 and T6 were similar across age groups. Persistence remained frequent in both groups (73.9% in participants aged ≥45 years vs. 70.0% in those aged <45 years) (Table S4). Incident HR-HPV infection at T6 was detected in participants from both age categories (24.1% in those aged ≥45 years vs. 14.3% in those aged <45 years), with no statistically significant difference between groups (OR 1.91, 95% CI: 0.40–10.09; *p* = 0.6934). The majority of baseline HR-HPV–negative individuals remained HR-HPV-negative at both visits regardless of age (Table S4).

A comparable pattern was observed for LR-HPV genotypes. Clearance occurred in 37.5% of participants aged ≥45 years and 50.0% of those younger than 45 years (OR 0.60, 95% CI: 0.11–3.32; *p* = 0.6550), with persistence rates of 62.5% and 50.0%, respectively (Table S4). LR-HPV incidence at T6 was detected in both age groups (13.9% vs. 11.1%), and most LR-HPV–negative participants remained negative at both T0 and T6, with similar proportions in older and younger individuals (86.1% vs. 88.9%; *p* > 0.9999).

#### 3.3.3. Dynamics of Vaccine-Type Oral HPV Infection by Age

In participants who were vaccine-type HPV-positive at T0, clearance at T6 was observed only in individuals aged ≥45 years (15.4%), whereas no clearance events were detected in participants younger than 45 years; persistence of vaccine-type HPV infection remained high in both age groups (84.6% vs. 100%) (Table S5).

For participants who were vaccine-type HPV-negative at T0, incident vaccine-type HPV infection at follow-up occurred at comparable rates in the two age groups (10.3% in participants aged ≥45 years and 11.1% in those aged <45 years; OR 0.91, 95% CI: 0.20–5.20; *p* > 0.9999). Consistently, most baseline-negative individuals remained negative for all vaccine-type HPV genotypes at both visits, irrespective of age (89.7% vs. 88.9%) (Table S5).

## 4. Discussion

In recent years, increasing evidence has suggested that prophylactic HPV vaccination is associated with a lower prevalence of oral HPV infection, particularly in younger vaccinated populations aged <26 years, both in women [28] and in men [29]. Beyond routine catch-up vaccination, the Advisory Committee on Immunization Practices (ACIP) recommends that HPV vaccination be considered through shared clinical decision-making for adults aged 27–45 years who are not adequately vaccinated [30]. This recommendation is particularly relevant for populations at increased risk of HPV acquisition and HPV-related disease, including gay, bisexual, and other men who have sex with men (GBMSM), transgender people, and individuals with immunocompromising conditions. This is especially important for GBMSM living with HIV, who have a higher burden of HPV-associated disease [31]. Similarly, HPV vaccination is strongly recommended by Canada’s National Advisory Committee on Immunization (NACI) for all MSM PLWH, regardless of age [32].

Moreover, the introduction of antiretroviral therapy (ART) has markedly increased life expectancy among PLWH. As a consequence, persistent HPV infections may become more clinically relevant over time, potentially contributing to the development of HPV-associated malignancies.

In this study, we evaluated the potential impact of the nine-valent HPV vaccine in a cohort of MSM PLWH, with a median age of 51 years (range: 30–70), and a large proportion of participants aged ≥45 years (68.4%). Importantly, all participants had initiated HPV vaccination after sexual debut. Vaccinated participants had completed the full three-dose schedule of the nine-valent HPV vaccine (Gardasil 9) at least two years before baseline sampling. Overall, immune status was stable, with a median CD4+ T-cell count of approximately 710 cells/µL, supporting the expectation of a sustained vaccine-induced response under ART [33,34].

At baseline, oral HPV positivity was high in the overall cohort (59.2%) and was numerically higher among unvaccinated than vaccinated participants (75.0% vs. 56.3%), although this difference was not statistically significant. HR-HPV genotypes predominated over LR-HPV genotypes at both time points, and HPV16 was the most frequently detected genotype overall, with a prevalence of 10.5% at T0 and 11.8% at T6. At baseline, HPV16 was followed by HPV73, HPV56, and HPV66, whereas at T6 the most frequent HR-HPV genotypes after HPV16 were HPV66, HPV33, HPV39, and HPV58.

When stratified according to vaccination status, HPV16 remained the most prevalent HR-HPV genotype among vaccinated participants, with a prevalence of 10.9% at T0 and 12.5% at T6. In contrast, among unvaccinated participants, HPV35 and HPV73 were the most frequently detected HR-HPV genotypes at both time points. HPV18 was not detected at T0 among vaccinated participants but was observed at T6, whereas in unvaccinated participants it was detected at both T0 and T6.

Among LR-HPV genotypes, HPV61 was consistently among the most frequent types at both T0 and T6 in vaccinated and unvaccinated participants, with higher baseline prevalence in the unvaccinated group. HPV53 was frequent only among vaccinated participants, while HPV44 emerged among the most common types at T6 in both groups.

Furthermore, age-stratified analyses indicated differences in genotype distribution, with HPV61 being the most frequently detected genotype in participants younger than 45 years, while HPV16 and HPV53 were the most common in participants aged 45 years or older.

In general, these prevalence estimates are higher than those reported in several studies of PLWH and MSM, as well as in the general population. For example, a recent meta-analysis among PLWH estimated a pooled oral HPV16 prevalence of approximately 4% [35]. Clinic-based studies in MSM PLWH have also reported lower overall oral HPV prevalence than that observed in the present cohort. In an Italian MSM cohort, oral HPV prevalence among MSM PLWH was 27.8%, with HPV16, HPV18, HPV72, and HPV84 among the most frequently detected genotypes (4.2% each) [36]. Similarly, studies conducted in the general population have reported substantially lower oral HPV16 prevalence estimates than those observed in our cohort [13,37].

The high baseline prevalence in the present study likely reflects, at least in part, differences in study populations and clinical settings, as well as the older age distribution of our participants. Our cohort had a median age of 51 years, with most participants aged ≥45 years. This is consistent with previous evidence showing that age is an important determinant of oral HR-HPV infection among MSM. In a study conducted in Mexico, Carnalla et al. reported a prevalence of any oral HR-HPV of 11.1% among MSM, increasing to 14.8% among men living with HIV; importantly, age ≥40 years was independently associated with oral HR-HPV infection (OR = 2.71, 95% CI: 1.28–5.73) [38]. In a previous clinic-based study performed at our center, oral HPV DNA was detected in 20.9% of PLWH, predominantly MSM, a prevalence markedly lower than that observed at baseline in the present study [39]. Similarly, in a large prospective analysis of MSM and transgender women undergoing nine-valent HPV vaccination, baseline oral HPV positivity was 14.2% [40].

The persistence of any HPV infection among baseline-positive participants in our cohort was substantially higher than the 24.3% persistence reported in the HPV Infection in Men (HIM) Study, although persistence in that study increased among individuals aged >39 years [16]. In another longitudinal Italian study of MSM PLWH, Parisi et al. reported confirmed oral HR-HPV detection in 40% of participants who were HR-HPV-positive at baseline over a 24-month follow-up, with persistence involving HPV16, HPV35, HPV56, and HPV59 [41].

These discrepancies may be explained by differences in sampling procedures, detection methods, follow-up intervals, and study population characteristics [42]. In particular, differences in age distribution and cohort composition may have contributed to the observed variability, as highlighted by Rossotti et al., whose study population included lower proportions of participants aged >45 years and MSM PLWH compared with the present cohort [40]. Moreover, our cohort was largely composed of participants who had been followed at our Institute for several years. Individuals with previous HPV positivity were routinely monitored and recalled for follow-up, which may have enriched the cohort for participants with prior or recurrent HPV detection and could partly explain the higher prevalence and persistence observed in the present study.

The only factor significantly associated with baseline oral HPV positivity in our cohort was the number of sexual partners in the previous year (*p* = 0.0298), in line with previous evidence linking oral HPV infection to sexual exposure [13,43].

Between T0 and T6, persistence of any HPV infection among those HPV-positive at baseline was identical in vaccinated and unvaccinated groups (77.8%), and clearance occurred at the same rate (22.2%). Persistence was also high for HR-HPV (69.2% in vaccinated vs. 85.7% in unvaccinated) and for LR-HPV (57.9% vs. 66.7%). To further characterize persistent HPV infections, viral burden dynamics were assessed using ΔΔCt values calculated between T6 and T0. Overall, ΔΔCt values were comparable between vaccinated and unvaccinated participants (median 0.21 vs. 0.25; *p* = 0.8810), despite substantial inter-individual variability. Genotype-specific patterns suggested that, among vaccinated participants, HPV16, as well as HPV33 and HPV82, tended to show stable or increasing viral loads over time, reflected by median ΔΔCt values below zero, whereas other genotypes showed decreasing viral loads. This pattern may indicate a greater likelihood of persistence for genotypes with negative ΔΔCt values and a reduced likelihood of persistence for those with positive ΔΔCt values. However, given the lack of standardization of this method, the analysis should be regarded as exploratory, and strong conclusions regarding viral load changes cannot be drawn.

A lower incidence of new infections was generally observed in our vaccinated participants, although not always statistically significant for any HPV over the short follow-up. Notably, when focusing on vaccine-type genotypes (HPV6, HPV11, HPV16, HPV18, HPV31, HPV33, HPV45, HPV52, and HPV58), incident vaccine-type infection among those negative at baseline was less frequent in vaccinated than in unvaccinated participants (6.3% vs. 33.3%; *p* = 0.0441). Importantly, this observational study was not designed to assess vaccine efficacy or effectiveness. However, this comparison was based on only three incident cases in each group and on a very small number of baseline-negative unvaccinated participants. Therefore, this finding should be considered hypothesis-generating rather than confirmatory and interpreted with caution. It may be consistent with a possible protective effect of vaccination against acquisition of vaccine-targeted oral HPV genotypes, even in adults vaccinated after sexual debut [44]. Conversely, among participants already positive for vaccine-type HPV at T0, persistence remained high in both groups (87.5% in vaccinated vs. 100.0% in unvaccinated), while clearance was infrequent and observed only among vaccinated participants (12.5%).

Age stratification did not reveal major differences in clearance or persistence for any HPV, HR-HPV, LR-HPV, or vaccine-type categories over the 6-month interval. Incident oral HPV infection among baseline-negative participants was numerically higher in the older group than in the younger group (40.0% vs. 18.2%), and vaccine-type HPV incidence was comparable between age groups.

The high prevalence and persistence of oral HPV observed in our study may partly reflect the aging of PLWH in the ART era, as increased life expectancy [45] may allow longer cumulative exposure to HPV and a greater likelihood of persistent infection. Beyond this demographic effect, a possible contribution of long-term antiretroviral exposure could be hypothesized, although this interpretation remains speculative. In vitro, Alam et al. showed that ritonavir may facilitate HPV16 infection [46]. Although ritonavir-containing regimens are less central to current ART strategies than in earlier treatment eras, many long-term PLWH, particularly older individuals, may have been exposed to protease inhibitor-based regimens over time. In our cohort, participants had a long history of HIV infection, especially those aged ≥45 years, and prior exposure to protease inhibitors, including darunavir-based regimens, could therefore be considered a potential contributing factor. However, this hypothesis cannot be confirmed within the design of the present study.

Consistent with this hypothesis, previous data showed that HPV-associated oral lesions in HIV patients were associated with older age (≥40 years) and prolonged HAART exposure (≥12 months), suggesting that their occurrence may be related to longer life expectancy in individuals with impaired immune function rather than to a direct effect of HAART [47]. Similarly, a meta-analysis reported a higher prevalence of HPV-related oral lesions among individuals receiving HAART compared with untreated subjects [48].

Several limitations in this study should be considered. The observational design and absence of randomization preclude causal inference and do not allow firm conclusions regarding vaccine efficacy or effectiveness. In addition, recruitment at a single referral center and inclusion of participants already engaged in long-term follow-up may have introduced selection bias and may limit generalizability.

The number of unvaccinated participants was small, limiting statistical power and the precision of effect estimates. In addition, small denominators in several subgroup analyses did not allow multivariable adjustment; therefore, residual confounding by age, sexual behavior, baseline HPV status, and other behavioral variables cannot be excluded. Follow-up was limited to 6 months; longer observation is needed to better characterize persistence and clearance in the oral cavity and to assess whether genotype-level viral load trajectories predict longer-term persistence. Vaccination occurred after sexual debut, and baseline vaccine-type HPV positivity was present in both vaccinated and unvaccinated participants, limiting conclusions regarding vaccine effects on prevalent infections and emphasizing the relevance of incident infection endpoints in adult cohorts. No immune response measurements were available, and oral lesions or other clinical endpoints were not systematically assessed during follow-up; therefore, the clinical relevance of oral HPV detection and persistence could not be evaluated.

Furthermore, the participant-level definition of persistence for HR-HPV, LR-HPV, and vaccine-type HPV was based on category-level positivity at both visits, regardless of genotype identity, which may overestimate true biological persistence.

Finally, several self-reported behavioral variables (sexual activity, alcohol consumption, smoking, and drug use) contained missing data. While self-reporting was used to reduce social-desirability bias, this missingness may have influenced the identification of HPV risk factors. Despite these limitations, this study provides prospective data on oral HPV dynamics in vaccinated adult MSM PLWH receiving ART. Importantly, the ongoing follow-up of this cohort may provide useful information on HPV persistence in vaccinated adults, particularly those aged ≥45 years, a population for whom longitudinal data remain limited.

## 5. Conclusions

Oral HPV prevalence and persistence were high in this cohort of MSM living with HIV and were broadly similar by vaccination status. However, vaccination was associated with a significantly lower incidence of vaccine-type oral HPV infection at follow-up (6.3% in vaccinated participants vs. 33.3% in unvaccinated participants; *p* = 0.0441). These findings support the rationale for offering HPV vaccination to adult populations at increased risk and underscore the need for larger studies with longer follow-up to better define the magnitude and durability of vaccine-associated protection at the oral site. Moreover, they highlight the importance of developing and promoting oral HPV surveillance and early-detection strategies, particularly in high-risk populations, given the absence of established routine screening programs for HPV-related oropharyngeal disease.

## Figures and Tables

**Table 1:** Baseline characteristics of the study population at baseline overall and stratified by HPV vaccination status (*N* = 76).

	Total (*n* = 76)	HPV-Vaccinated (*n* = 64)	HPV-Unvaccinated (*n* = 12)	OR (95% CI)	*p*
Age, median years (range) ≥45 years, *n* (%) <45 years, *n* (%)	51 (30–70) 52 (68.4) 24 (31.6)	51 (30–70) 43 (67.2) 21 (32.8)	48.5 (38–63) 9 (75.0) 3 (25.0)	0.68 (0.18–2.52)	0.7864 0.7422
Age at vaccine initiation, median years (range)		47 (26–68)			
Ethnicity, *n* (%) Italian Other	68 (89.5) 8 (10.5)	59 (92.2) 5 (7.8)	9 (75.0) 3 (25.0)	3.93 (0.90–20.23)	0.1075
HPV status, *n* (%) HPV-positive HPV-negative	45 (59.2) 31 (40.8)	36 (56.3) 28 (43.7)	9 (75.0) 3 (25.0)	0.43 (0.12–1.55)	0.3395
Multiple HPV infection, *n* (%)	19 (25.0)	15 (23.4)	4 (33.3)	0.61 (0.17–2.05)	0.4810
Positive for at least one HR-HPV genotype, *n* (%)	33 (43.4)	26 (40.6)	7 (58.3)	0.49 (0.15–1.67)	0.3445
Positive for at least one HPV genotype included in the nine-valent vaccine, *n* (%)	19 (25.0)	16 (25.0)	3 (25.0)	1 (0.26–3.77)	>0.9999
HIV RNA, *n* (%) Not detected Detectable: <30 copies/mL >30 copies/mL	49 (64.5) 26 (34.2) 1 (1.3)	41 (64.1) 22 (34.3) 1 (1.6)	8 (66.6) 4 (33.3)0	0.89 (0.27–3.05)	>0.9999
CD4+ T cell count, median cells/μL (IQR) ≥500/μL, *n* (%) 201–499/μL, *n* (%) ≤200/μL, *n* (%)	710.0 (504.0–895.0) 59 (77.7) 15 (19.7) 2 (2.6)	713.0 (515.0–889.5) 51 (79.6) 12 (18.8) 1 (1.6)	672.0 (288.5–884.0) 8 (66.7) 3 (25.0) 1 (8.3)		0.5331

**Table 2:** Univariable analysis of factors associated with baseline oral HPV positivity in the overall study population (*N* = 76).

Characteristic	HPV Positivity *N* = 45	HPV Negativity *N* = 31	OR (95% CI)	*p*
Age				
≥45 years	32	20	1.35 (0.48–3.71)	0.6190
<45 years	13	11		
CD4+ T-cell count				
≥500	37	23	1.61 (0.54–4.59)	0.4087
<500	8	8		
HIV RNA				
detectable	16	11	1 (0.39–2.75)	>0.9999
undetectable	29	20		
HPV vaccination				
Yes	36	28	0.43 (0.12–1.55)	0.3395
No	9	3		
Sexual partners in the last year *				
≥10	23	5	4.09 (1.33–11.85)	0.0298
<10	18	16		
Practiced oral sex *				
Often	32	19	0.79 (0.26–2.70)	0.7710
Never/rarely	10	5		
Received oral sex *				
Often	29	17	0.92 (0.33–2.63)	>0.9999
Never/rarely	13	7		
Smoking *				
Yes	18	9	1.20 (0.41–3.35)	0.7988
No	25	15		
Regular alcohol consumption *				
Yes	11	9	0.57 (0.20–1.72)	0.4050
No	32	15		
Recreational drug use *				
Yes	9	6	0.79 (0.26–2.82)	0.7643
No	34	18		

* Behavioral variables include missing data due to incomplete questionnaire responses. Missing values were as follows: sexual partners in the last year, *n* = 14; practiced oral sex, *n* = 10; received oral sex, *n* = 10; smoking, *n* = 9; regular alcohol consumption, *n* = 9; recreational drug use, *n* = 9.

**Table 3:** Oral HPV dynamics between T0 and T6 by vaccination status.

Outcome (T0→T6)	HPV-Vaccinated *n*/*N* (%)	HPV-Unvaccinated *n*/*N* (%)	OR (95% CI)	*p*
Clearance of any HPV T0+ → T6−	8/36 (22.2)	2/9 (22.2)	1 (0.19–5.51)	>0.9999
Persistence of any HPV T0+ → T6+	28/36 (77.8)	7/9 (77.8)		
Incidence of any HPV T0− → T6+	8/28 (28.6)	2/3 (66.7)	0.20 (0.02–2.53)	0.2369
Remaining negative T0− → T6−	20/28 (71.4)	1/3 (33.3)		

T0+, presence of at least one HPV genotype at baseline; T0−, absence of any HPV genotype at baseline; T6+, presence of at least one HPV genotype at follow-up (6 months); T6−, absence of any HPV genotype at follow-up (6 months).

**Table 4:** Dynamics of high-risk and low-risk HPV genotype oral infections between T0 and T6 by vaccination status.

Outcome (T0→T6)	HPV-Vaccinated *n*/*N* (%)	HPV-Unvaccinated *n*/*N* (%)	OR (95% CI)	*p*
High-risk HPV genotype				
Clearance HR+ → HR−	8/26 (30.8)	1/7 (14.3)	2.67 (0.28–34.01)	0.6418
Persistence HR+ → HR+	18/26 (69.2)	6/7 (85.7)		
Incidence HR− → HR+	7/38 (18.4)	2/5 (40.0)	0.34 (0.06–2.25)	0.2773
No HR-HPV at both visits HR− → HR−	31/38 (81.6)	3/5 (60.0)		
Low-risk HPV genotype				
Clearance LR+ → LR−	8/19 (42.1)	1/3 (33.3)	1.46 (0.15–23.43)	>0.9999
Persistence LR+ → LR+	11/19 (57.9)	2/3 (66.7)		
Incidence LR− → LR+	6/45 (13.3)	1/9 (11.1)	1.23 (0.14–15.73)	>0.9999
No LR-HPV at both visits LR− → LR−	39/45 (86.7)	8/9 (88.9)		

HR+, presence of at least one high-risk genotype; HR−, absence of any high-risk genotype; LR+, presence of at least one low-risk genotype; LR−, absence of any low-risk genotype.

**Table 5:** Dynamics of vaccine-type HPV infection between T0 and T6 by vaccination status.

Outcome (T0→T6)	HPV-Vaccinated *n*/*N* (%)	HPV-Unvaccinated *n*/*N* (%)	OR (95% CI)	*p*
Clearance of vaccine-type HPV vaccine-type+ → vaccine-type−	2/16 (12.5)	0/3 (0.0)	NE	>0.9999
Persistence of vaccine-type HPV vaccine-type+ → vaccine-type+	14/16 (87.5)	3/3 (100.0)		
Incidence of vaccine-type HPV vaccine-type− → vaccine-type+	3/48 (6.3)	3/9 (33.3)	0.13 (0.03–0.71)	0.0441
No vaccine-type HPV at both visits vaccine-type− → vaccine-type−	45/48 (93.7)	6/9 (66.7)		

Vaccine-type+, participants positive for at least one vaccine-type HPV genotype (HPV6, HPV11, HPV16, HPV18, HPV31, HPV33, HPV45, HPV52, and HPV58); vaccine-type−, participants negative for all vaccine-type HPV genotypes; NE, not estimable because no clearance events were observed in the unvaccinated group, resulting in a zero cell count and preventing reliable estimation of the odds ratio.

## Data Availability

The data presented in this study are available on request from the corresponding author.

## Supplementary Materials

The following supporting information can be downloaded at: https://www.mdpi.com/article/10.3390/vaccines14070589/s1, Table S1: Prevalence of oral HPV genotypes at baseline (T0); Table S2: Prevalence of oral HPV genotypes at follow-up (T6); Table S3: Oral HPV dynamics between T0 and T6 by age; Table S4: Dynamics of high-risk and low-risk HPV genotype oral infections between T0 and T6 by age; Table S5: Dynamics of vaccine-type HPV infection between T0 and T6 by age; Figure S1: Genotype-level distribution of oral HPV outcomes between baseline (T0) and 6-month follow-up (T6), stratified by vaccination status; Figure S2: Baseline (T0) ΔCt values in detections that persisted versus cleared at 6-month follow-up (T6).
