# Supplementary material for: Oral HPV Dynamics in MSM Living with HIV in the Nine-Valent HPV Vaccination Era
Source: Vaccines (Basel). 2026 Jul 1;14(7):589. doi: 10.3390/vaccines14070589 (PMC13417218; doi:10.3390/vaccines14070589)
Supplement: Supplementary file 1 [file vaccines-14-00589-s001.zip › vaccines-4347756-supplementary.pdf]

Table S1. Prevalence of oral HPV genotypes at baseline (T0)

**Genotype**	**Overall (N=76)**		**HPV-vaccinated (n=64)**		**HPV-unvaccinated (n=12)**	
	T0		T0		T0	
High-risk HPV	n	%	n	%	n	%
**16**	8	10.5%	7	10.9%	1	8.3%
**18**	1	1.3%	0	0.0%	1	8.3%
**31**	0	0.0%	0	0.0%	0	0.0%
**33**	3	3.9%	3	4.7%	0	0.0%
35	3	3.9%	1	1.6%	2	16.7%
39	4	5.3%	4	6.3%	0	0.0%
**45**	0	0.0%	0	0.0%	0	0.0%
51	1	1.3%	1	1.6%	0	0.0%
**52**	1	1.3%	1	1.6%	0	0.0%
56	5	6.6%	4	6.3%	1	8.3%
**58**	3	3.9%	2	3.1%	1	8.3%
59	2	2.6%	1	1.6%	1	8.3%
66	5	6.6%	4	6.3%	1	8.3%
68	0	0.0%	0	0.0%	0	0.0%
73	6	7.9%	4	6.3%	2	16.7%
82	3	3.9%	3	4.7%	0	0.0%
Low-risk HPV						
**6**	3	3.9%	3	4.7%	0	0.0%
**11**	2	2.6%	2	3.1%	0	0.0%
26	1	1.3%	1	1.6%	0	0.0%
40	0	0.0%	0	0.0%	0	0.0%
42	1	1.3%	1	1.6%	0	0.0%
43	1	1.3%	1	1.6%	0	0.0%
44	2	2.6%	1	1.6%	1	8.3%
53	7	9.2%	7	10.9%	0	0.0%
54	0	0.0%	0	0.0%	0	0.0%
61	8	10.5%	5	7.8%	3	25.0%
69	1	1.3%	1	1.6%	0	0.0%
70	2	2.6%	2	3.1%	0	0.0%
74	1	1.3%	1	1.6%	0	0.0%

HPV genotypes included in the nine-valent vaccine are shown in bold.

Table S2. Prevalence of oral HPV genotypes at follow-up (T6)

**Genotype**	**Overall (N=76)**		**HPV-vaccinated (n=64)**		**HPV-unvaccinated (n=12)**	
	T6		T6		T6	
High-risk HPV	n	%	n	%	n	%
**16**	9	11.8%	8	12.5%	1	8.3%
**18**	2	2.6%	1	1.6%	1	8.3%
**31**	0	0.0%	0	0.0%	0	0.0%
**33**	5	6.6%	4	6.3%	1	8.3%
35	2	2.6%	0	0.0%	2	16.7%
39	5	6.6%	5	7.8%	0	0.0%
**45**	1	1.3%	1	1.6%	0	0.0%
51	2	2.6%	2	3.1%	0	0.0%
**52**	0	0.0%	0	0.0%	0	0.0%
56	0	0.0%	0	0.0%	0	0.0%
**58**	5	6.6%	4	6.3%	1	8.3%
59	1	1.3%	0	0.0%	1	8.3%
66	6	7.9%	5	7.8%	1	8.3%
68	2	2.6%	2	3.1%	0	0.0%
73	3	3.9%	1	1.6%	2	16.7%
82	3	3.9%	3	4.7%	0	0.0%
Low-risk HPV						
**6**	3	3.9%	1	1.6%	2	16.7%
**11**	3	3.9%	3	4.7%	0	0.0%
26	0	0.0%	0	0.0%	0	0.0%
40	1	1.3%	1	1.6%	0	0.0%
42	0	0.0%	0	0.0%	0	0.0%
43	3	3.9%	3	4.7%	0	0.0%
44	6	7.9%	4	6.3%	2	16.7%
53	4	5.3%	4	6.3%	0	0.0%
54	1	1.3%	1	1.6%	0	0.0%
61	5	6.6%	3	4.7%	2	16.7%
69	1	1.3%	1	1.6%	0	0.0%
70	2	2.6%	2	3.1%	0	0.0%
74	0	0.0%	0	0.0%	0	0.0%

HPV genotypes included in the nine-valent vaccine are shown in bold.

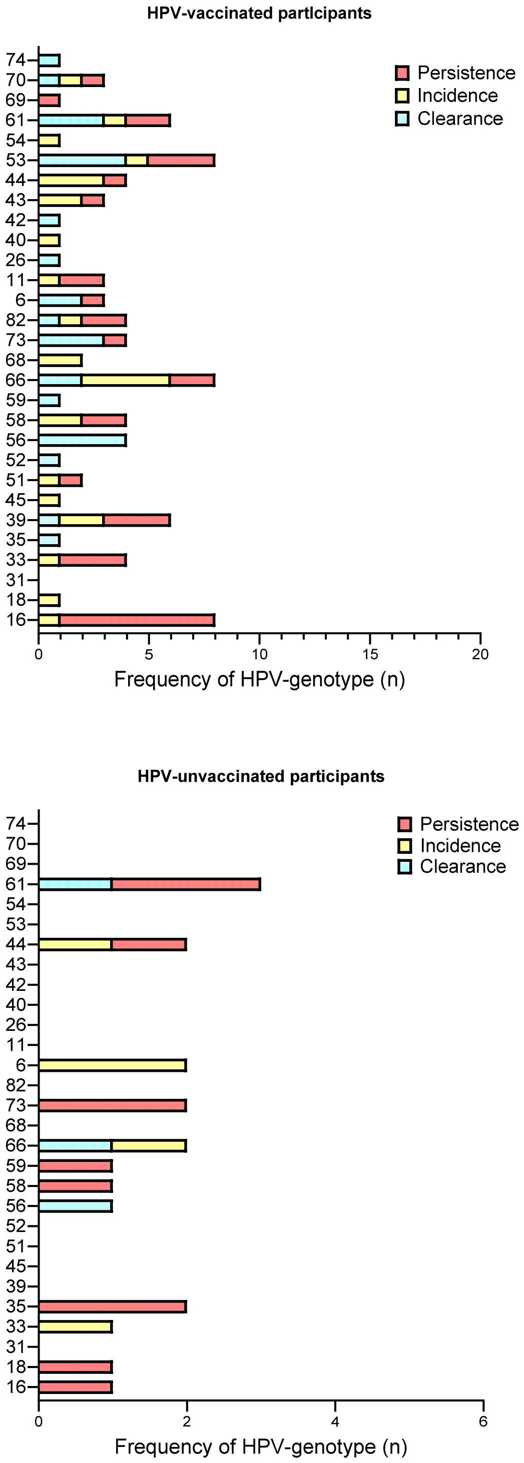

Figure S1. Genotype-level distribution of oral HPV outcomes between baseline (T0) and 6-month follow-up (T6), stratified by vaccination status. Each horizontal bar represents a specific HPV genotype and shows the number of genotype-specific events classified as persistence (red), incidence (yellow), or clearance (blue) from T0 to T6. Counts reflect genotype-specific detections; participants could contribute more than one genotype-specific event.

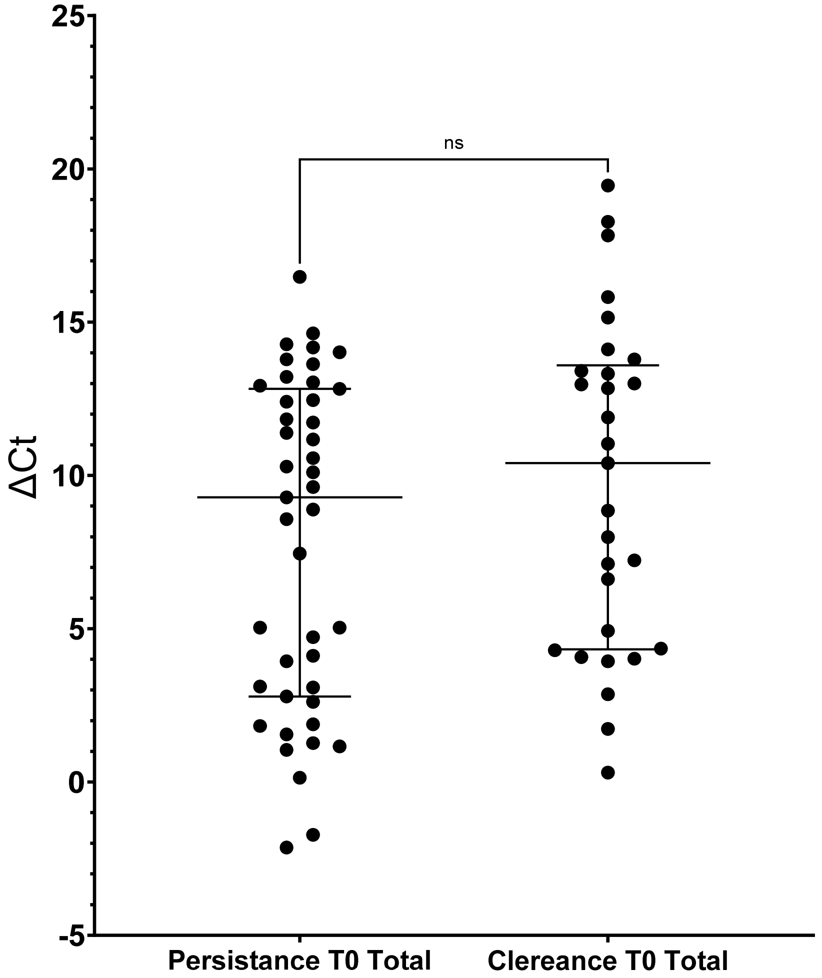

Figure S2. Baseline (T0) ΔCt values in detections that persisted versus cleared at 6-month follow-up (T6). Each dot represents a genotype-specific detection. Horizontal lines indicate the median with interquartile range (IQR). ns, not significant ($p = 0.167$).

Table S3. Oral HPV dynamics between T0 and T6 by age

Outcome (T0→T6)	≥ 45 years n/N (%)	< 45 years n/N (%)	OR (95% CI)	*p*
Clearance of any HPV (T0+ → T6−)	7/32 (21.9)	3/13 (23.1)	0.93 (0.23-3.84)	>0.9999
Persistence of any HPV (T0+ → T6+)	25/32 (78.1)	10/13 (76.9)		
Incidence of any HPV (T0− → T6+)	8/20 (40.0)	2/11 (18.2)	3.00 (0.56-16.19)	0.2617
Remaining negative (T0− → T6−)	12/20 (60.0)	9/11 (81.8)		

T0+, presence of at least one HPV genotype at baseline; T0−, absence of any HPV genotype at baseline; T6+, presence of at least one HPV genotype at follow-up (6 months); T6−, absence of any HPV genotype at follow-up (6 months).

Table S4. Dynamics of high-risk and low-risk HPV genotype oral infections between T0 and T6 by age

Outcome (T0→T6)	≥ 45 years n/N (%)	< 45 years n/N (%)	OR (95% CI)	*p*
High-risk HPV genotype				
Clearance (HR+ → HR−)	6/23 (26.1)	3/10 (30.0)	0.69 (0.14-3.19)	0.6808
Persistence (HR+ → HR+)	17/23 (73.9)	7/10 (70.0)		
Incidence (HR− → HR+)	7/29 (24.1)	2/14 (14.3)	1.91 (0.40–10.09)	0.6934
No HR-HPV at both visits (HR− → HR−)	22/29 (75.9)	12/14 (78.6)		
Low-risk HPV genotype				
Clearance (LR+ → LR−)	6/16 (37.5)	3/6 (50.0)	0.60 (0.11-3.32)	0.6550
Persistence (LR+ → LR+)	10/16 (62.5)	3/6 (50.0)		
Incidence (LR− → LR+)	5/36 (13.9)	2/18 (11.1)	1.29 (0.22-7.02)	>0.9999
No HR-HPV at both visits (LR− → LR−)	31/36 (86.1)	16/18 (88.9)		

HR+, presence of at least one high-risk genotype; HR-, absence of any high-risk genotype; LR+, presence of at least one low-risk genotype; LR-, absence of any low-risk genotype.

Table S5. Dynamics of vaccine-type HPV infection between T0 and T6 by age

Outcome (T0→T6)	≥ 45 years n/N (%)	< 45 years n/N (%)	OR (95% CI)	*p*
Clearance of vaccine-type HPV vaccine-type (T0) + →vaccine-type (T6) −	2/13 (15.4)	0/6 (0.0)	NE	>0.9999
Persistence of vaccine-type HPV vaccine-type (T0) + →vaccine-type (T6) +	11/13 (84.6)	6/6 (100)		
Incidence of vaccine-type HPV vaccine-type (T0) − →vaccine-type (T6) +	4/39 (10.3)	2/18 (11.1)	0.91 (0.20-5.20)	>0.9999
No vaccine-type HPV at both visits vaccine-type (T0) − →vaccine-type (T6) −	35/39 (89.7)	16/18 (88.9)		

Vaccine-type+, participants positive for at least one vaccine-type HPV genotype (HPV6, HPV11, HPV16, HPV18, HPV31, HPV33, HPV45, HPV52, and HPV58); vaccine-type -, participants negative for all vaccine-type HPV genotypes; NE, not estimable.
